# Lineages of varicella-zoster virus

**DOI:** 10.1099/vir.0.007658-0

**Published:** 2009-04

**Authors:** Duncan J. McGeoch

**Affiliations:** Medical Research Council Virology Unit, Institute of Virology, University of Glasgow, Church Street, Glasgow G11 5JR, UK

## Abstract

Relationships among varicella-zoster virus (VZV; *Human herpesvirus 3*) genome sequences were examined to evaluate descent of strains, structures of lineages and incidence of recombination events. Eighteen complete, published genome sequences were aligned and 494 single nucleotide polymorphisms (SNPs) extracted, each as two alleles. At 281 SNPs, a single sequence differed from all the others. Distributions of the remaining 213 SNPs indicated that the sequences fell into five groups, which coincided with previously recognized phylogenetic groupings, termed E1, E2, J, M1 and M2. The 213-SNP set was divisible into 104 SNPs that were specific to a single group, and 109 cross-group SNPs that defined relationships among groups. This last set was evaluated by criteria of continuities in relationships between groups and breaks in such patterns, to identify crossover points and ascribe them to lineages. For the 99 cross-group SNPs in the genome's long unique region, it was seen that the E2 and M2 groups were almost completely distinct in their SNP alleles, and the E1 group was derived from a recombinant of E2 and M2. A valid phylogenetic tree could thus be constructed for the four E2 and two M2 strains. There was no substantive evidence for recombination within the E2 group or the E1 group (ten strains). The J and M1 groups each contained only one strain, and both were interpreted as having substantial distinct histories plus possible recombinant elements from the E2 and M2 lineages. The view of VZV recombination and phylogeny reached represents a major clarification of deep relationships among VZV lineages.

## INTRODUCTION

Varicella-zoster virus (VZV) (subfamily *Alphaherpesvirinae*, genus *Varicellovirus*, species *Human herpesvirus 3*) is the causative agent of chickenpox and shingles, and is one of eight herpesviruses that have humans as the natural host. VZV has a double-stranded DNA genome of 125 kbp, which is the smallest among the human herpesviruses (Davison & Scott, 1986). VZV also exhibits a notably low genomic diversity among isolates, relative to other studied herpesviruses (Quinlivan & Breuer, 2006). The first complete genomic sequence, for the Dumas strain, was published in 1986 (Davison & Scott, 1986), and sequences are now available for 18 isolates (see Table 1). It is apparent that the sequences fall into several groups, and distinct patterns of association of groups in different genomic regions indicate that recombination must have played a role in generation of at least some of the present-day groups (Norberg *et al.*, 2006; Peters *et al.*, 2006; Tyler *et al.*, 2007). VZV strains have also been characterized by more limited DNA sequencing, with applications to molecular epidemiology of wild-type strains and to investigating behaviour of vaccine strains in human populations (Quinlivan & Breuer, 2006; Sauerbrei *et al.*, 2006).

**Fig. 1. f1:**
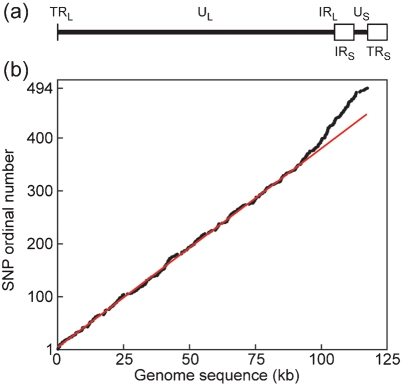
VZV genomic organization and distribution of SNPs. (a) Genomic layout of VZV, with U_L_ and U_S_ as heavy lines, IR_S_ and TR_S_ as open boxes, and the locations of the tiny (88 bp) TR_L_ and IR_L_ elements indicated. (b) Distribution of 494 SNPs on the genome, with genomic location (confluent black dots) plotted against SNP ordinal number. The red line is constructed as the best straight line through SNPs 1–350. (a) and (b) are in register.

VZV genomic DNA (in the linear form found in virions) is treated as consisting of two linked components, the long (L) and short (S) regions. Each of these contains a unique sequence (U_L_ and U_S_, 105 and 5.2 kbp, respectively) flanked by a pair of repeat elements in opposing orientations: R_L_ (88 bp) and R_S_ (7.3 kbp). Terminal and internal copies of the repeats are denoted by the prefixes T and I respectively, and the complete DNA molecule then has the arrangement TR_L_-U_L_-IR_L_-IR_S_-U_S_-TR_S_ (Dumas *et al.*, 1981; Davison & Scott, 1986), as shown in Fig. 1(a). There are two primary isomeric forms of the genome, occurring at equivalent frequencies, which differ in the orientation of U_S_ relative to U_L_, and one of these is designated the prototype. In addition, there are two further, lower frequency, isomeric forms in which U_L_ is in the other orientation relative to U_S_ (Davison, 1984). The copies of R_L_ and R_S_ are regarded as being maintained in a homogeneous state by recombination.

This paper is concerned with deriving, from available genomic sequence data, a view of the evolutionary development of VZV lineages. The low diversity of VZV sequences and the occurrence of recombination are limiting factors in such work, and previous analyses have not, in my view, reached the limits of objective inference. The 18 genome sequences now available provide a dataset of reasonable size and power, which I have utilized to obtain insights into recombination events and their localization to specific lineages, and to construct an appropriate phylogenetic representation of the descent of VZV lineages and isolates.

## METHODS

VZV genome sequences were obtained from GenBank (Table 1). Sequences were aligned using mafft (Katoh *et al.*, 2002), and manipulations carried out using text editors and the gcg (Accelrys) and emboss program suites. Phylogenetic trees were constructed with programs dnapars and pars of the phylip suite (Felsenstein, 2005), and by Bayesian Monte Carlo Markov Chain (BMCMC) analysis with the program MrBayes (Ronquist & Huelsenbeck, 2003). Figures were composed in PostScript (Adobe).

## RESULTS

### Derivation of VZV genomic polymorphism sets

As of July 2008 there were 23 complete genomic sequences for VZV strains available. Three are for vaccine strains and two are for high-passage versions of a separately sequenced strain, and these five were omitted from the analysis described here. An alignment was constructed with the remaining 18 DNA sequences (listed in Table 1). Before use for analyses of relationships, cleaning procedures were carried out as follows. First, the terminal copies of repeat elements R_L_ and R_S_ were removed, thus leaving only the internal copy of each. Second, sites of apparent or suspected high-frequency variation were removed, specifically tandem reiterations or simple sequences, the origin of DNA replication and the joint between the L and S segments. Third, loci with a gapping character in any sequence were deleted. The resulting alignment comprised 116.5 kb. It should be noted that U_L_ and U_S_ should be treated as unlinked because of the occurrence of orientation isomers, and that existence of two copies of R_S_ means that R_S_ and U_S_ are not linked simply. Analyses used exclusively single nucleotide polymorphisms (SNPs), there being very few potentially useful insertion–deletion polymorphisms. The alignment contained 494 SNPs, all of which were present in only two allelic states. The distribution of SNPs across the genome is illustrated in Fig. 1(b), which plots the genomic location of each against its ordinal number (that is, 1–494). At the low resolution of this plot the SNPs appear as evenly distributed, except for a region of higher incidence near the right end of the genome. This region includes the 7.3 kb IR_S_, and it is entirely credible that higher levels of variation should be associated with this extended repeat element. However, the region of higher SNP incidence clearly also encompasses several thousand residues of the right extremity of U_L_, and here no ready explanation is apparent.

**Fig. 2. f2:**
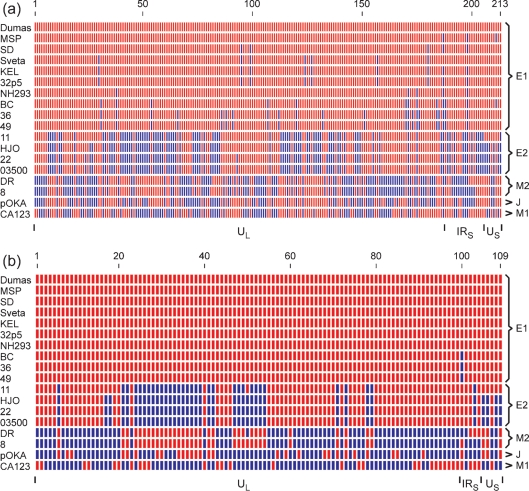
Relationships of non-singleton SNPs. For each of the 18 VZV genomes, SNPs are shown coloured red where identical to the Dumas allele at the same locus, and blue where different from the Dumas allele. (a) The set of 213 non-singleton SNPs. (b) The set of 109 cross-group SNPs. For both panels, genomic regions to which the SNPs belong (U_L_, IR_S_ and U_S_) are indicated at the bottom and memberships of the E1, E2, M2, J and M1 groups at the right.

Of the 494 SNPs, 281 represent loci at which one sequence differs from the other 17. Such singleton diversity will in most cases define the extent to which a single isolate has changed since the line leading to it diverged from the most closely related line or lines (a terminal branch that contained the tree's root would constitute a special case, which I consider does not occur in the VZV dataset). Singleton SNPs are not of primary relevance for analysing deeper relationships among lineages. Alleles for the 213 non-singleton SNPs are illustrated in Fig. 2(a), in which each SNP locus in each strain was coloured to indicate identity or non-identity to the corresponding locus in the Dumas strain. Inspection showed that 16 of the strains fall easily into three major groups. The uppermost ten sequences as listed in Fig. 2(a) are close to the Dumas strain, the next four are close to HJO, and the next two (strains DR and 8) form a third group. The last two sequences, for the pOKA and CA123 strains, have distinct SNP patterns and were each treated as a separate group. The five groups defined in this way coincide with groups that had previously been recognized through tree-building comparisons of VZV genomic sequences (Norberg *et al.*, 2006; Peters *et al.*, 2006; Loparev *et al.*, 2007). Several nomenclatures for these groups have been used in published papers and that of Loparev *et al.* (2007) is followed here, as listed in Table 1.

Recognition of five groups by the criterion of SNP patterns then allowed division of the non-singleton SNPs into cross-group SNPs (which define deep relationships among groups) and group-specific SNPs. This last subset was taken to include both SNPs that define variation only within a single group, and also SNPs common to all members of a given group and distinct from all strains in other groups. There are 109 cross-group SNPs, which are depicted in Fig. 2(b), again coloured relative to the Dumas strain. Of the cross-group SNPs, 99 are in U_L_, five in IR_S_ and five in U_S_. IR_L_ contains only two SNPs, both singletons, and so does not appear in the analysis. Within U_L_, only three cross-group SNP loci are also variable within groups, namely SNPs 17 and 18 (from Fig. 2b[Fig f2]) in the HJO-like or E2 group, and SNP 50 in both the E2 group and the DR-like or M2 group. However, four of the five cross-group SNP loci in IR_S_, and also four of the five in U_S_, are variable within groups.

### Analysis of VZV genomic lineages and crossovers

It is apparent from Fig. 2(b) that over the cross-group set there exist regions both of extended identity and extended difference between groups, and that patterns of inter-group relationships vary along the genome. I wished to interpret the cross-group SNP set in terms of underlying lineages and of recombinational breakpoints assigned to particular groups. Before describing how this was approached, some comments on the dataset are needed. The SNPs are a binary dataset, so that at most two different lineages can be presented as each being uniformly coloured across a given region. It is implicit that at each SNP locus one allele represents the ancestral state and the other a substitution mutation. Since there are five groups and the dataset was constructed to exclude singletons, every cross-group SNP locus must show one allele in three groups and the other in two groups (excepting the few that show intra-group variability).

**Fig. 3. f3:**
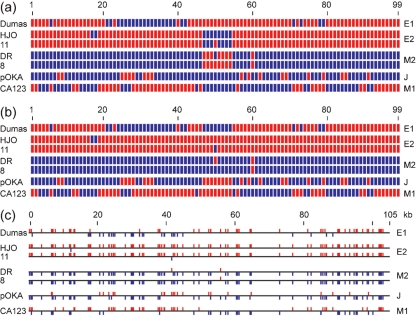
Optimized colouring schemes for cross-group SNPs in U_L_. (a) and (b) Two alternative optimized colouring schemes for the set of 99 cross-group SNPs in U_L_ were derived as described in the text, and are presented after the manner of Fig. 2, but with assignments of colours as obtained by the optimizing process. For the E1 group, all cross-group SNP sets in U_L_ are identical so only one sequence is shown, while for the E2 and M2 groups, intra-group variations require that two sequences are shown. (c) The colouring of the version shown in (b) is shown transferred to the actual genomic locus for each SNP.

Colouring SNPs by reference to any one strain is of course an arbitrary device (and indeed the colouring scheme in Fig. 2(b) is spuriously suggestive of a notably unlikely scenario: it gives the E2 and M2 groups the appearance of being complex reciprocal recombinants). I attempted to derive a colouring scheme that optimized visible continuity of lineages in terms of extended identity or difference between groups' SNPs. This exercise focused on U_L_, since the ten cross-group SNPs associated with IR_S_ and U_S_ showed high intra-group variation. In order to facilitate recognition of patterns in the array, the SNP set for the five groups was converted to binary form with alleles at each locus coded as 0 and 1 (equivalently to the colouring in Fig. 2b[Fig f2]). Inspection showed that the M1 group was an outlier in terms of defining patterns at single SNP loci that were present at high frequency. Single loci patterns were then examined for the other four groups, showing that the majority of loci for E1 were identical to those of E2 and most of those of M2 were identical to J. The SNP loci that differed from the majority pattern were individually tested for whether reversing their assignments of 0 and 1 symbols would improve net continuity of coding with the neighbouring SNPs, as summed over all five groups. A fuller account is given in the supplementary material available with the online version of this paper. Two optimized colouring schemes were obtained, which differ only in the region of SNPs 47–54; these are shown in Fig. 3(a and b). This procedure reduced the number of colour-change boundaries along the U_L_ cross-group SNP array, summed over the five groups (with intra-group variations averaged), from 95 for Dumas-relative colouring to 65 for both optimized schemes. The version in Fig. 3(b) is used for further discussion because of the general uniformity of colouring it achieves within both E2 and M2 groups (while noting that this was not a planned criterion in the optimization protocol). Fig. 3(c) shows how the scheme of Fig. 3(b) transfers to a genomic map of SNP loci.

The colouring scheme of Fig. 3(b) enabled a coherent interpretation of relationships in U_L_ among the five groups. The most cogent feature is that the E2 and M2 groups are almost completely composed of distinct alleles, and each of these groups can be regarded as comprising deep lineages that contain little or no admixture from recombination with other lineages. Next, the E1 group's alleles match those of the E2 group for the most part, but there are also substantial sections that match the M2 group (numbers 24–39 and 47–54). The E1 group thus appears to have descended from a recombinant between the E2 and M2 lineages. For the larger M2-like region in E1 (SNPs 24–39) this interpretation is strong with respect to the criteria used to derive the colour scheme: either the E1 lineage is recombinant at this locus and all four other lineages are not, or vice versa. For the smaller M2-like region (SNPs 47–54) the colouring choice is balanced. Smaller regions of heterogeneity of colours in E1, E2 and M2 could represent point mutational or recombinational events, whose SNP signatures are too meagre to be robustly interpretable. Lastly, the single-member J and M1 groups each have large single-coloured sections (for both colours) and also other heterogeneous sections. They thus seem to contain elements that are recombinant between the E2 and M2 lineages, while the patchy, mixed sections may, at least in part, represent deep lineages distinct from those of E2 and M2. Interpretation for J and M1 was limited by the absence of opportunity to refine their SNP sets through identifying variations specific to group or strain.

### Representation of phylogenetic relationships among VZV U_L_ sequences

In no case do the SNPs that define variation within a multi-member group show any substantive indication of recombination, and a valid phylogenetic tree for each group can thus be made showing relationships among members. However, given the clear indication that crossovers were involved in the genesis of at least some of these contemporary groups, a standard phylogenetic tree that includes all groups would not provide an accurate representation of deep relationships among groups. In this section I use the analyses of SNP data described above to reach a representation that takes recombinational events into account, based on the U_L_ sequences.

**Fig. 4. f4:**
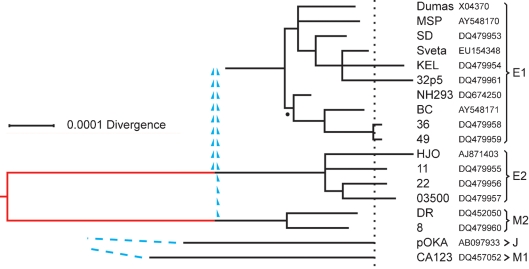
Phylogenetic relationships among VZV U_L_ sequences. BMCMC was used to derive a phylogenetic tree for the six sequences in the E2 plus M2 groups, and a separate, partial tree for the ten E1 sequences. The branching structures shown for the E1 and E2 groups are majority-rule trees, in which all resolved nodes had estimated posterior probabilities of 1.00 except for the E1 node marked with a dot, which had a posterior probability of 0.69. The red section in the E2–M2 tree corresponds to the contribution of the cross-group SNPs, and the tree was rooted at the midpoint of this section. The dotted line represents the positions of average tip loci between the E2 and M2 groups. Branch lengths for the J and M1 groups correspond to their singleton SNP sets. For E1, J and M1 groups, mean loci for the tips of terminal branches are placed on the dotted line. The recombinational origins of the E1 group from the E2 and M2 lineages are indicated by blue arrowheads, and the unresolved deep connections of the J and M1 groups by blue dashed lines. The scale bar indicates substitutions per site in the U_L_ alignment.

Since the U_L_ cross-group SNPs for the E2 and M2 groups are almost completely distinct, a tree based on only these two groups should be nearly free of any complications due to recombination. Trees were derived using the complete U_L_ alignments for the E2 and M2 strains. Initial work used maximum-parsimony programs, which allowed the contributions of the several subsets of SNPs to be readily distinguished, and this was then supplemented by the computationally more sophisticated BMCMC approach. The two methods gave effectively identical results. Because diversity in the genomic alignment is low, allowance for multiple substitutions made by the BMCMC computation is small; the overall branch length of the BMCMC tree was less than 2 % greater than that of its maximum-parsimony counterpart. The tree for E2 plus M2 from BMCMC is shown in Fig. 4, with the section that represents divergence attributable to cross-group SNPs drawn in red. In the case of the E1 group, a tree was derived using the complete E1 and M2 U_L_ alignments, and treating M2 as outgroup for E1. This E1 tree is valid for the portion corresponding to the E1-group-specific SNPs; that is, for descent from the earliest group-specific ancestor. However, since it appears that the lineage ancestral to E1 arose by recombination events involving the E2 and M2 lineages, the deeper history of the E1 group can only be indicated by sketched connections to those lineages, as depicted in Fig. 4. It is to be noted that the recombinational genesis of the E1 line evidently took place later than most of the substitutional divergence events defined by the cross-group SNP configurations of the E2 and M2 groups. Lastly, the single-member J and M1 groups each possess unique lineages of substantial relative depth, as indicated by their singleton SNP complements, while the inferred existence of crossover events obscures details of their deeper connections (Fig. 4).

## DISCUSSION

The view of VZV recombination and phylogeny achieved in this paper represents a major clarification in our appreciation of relationships among VZV lineages. While the sequences are highly conserved, there is sufficient diversity in the set of 18 genomes analysed to define major lineages and to give some insight into possible crossover events. Earlier phylogenetic analyses of whole genome sequences had shown that five major groups or clades could be discerned, but that the detail of relationships among these varied along the genome, indicating the presence of recombinant forms (Norberg *et al.*, 2006; Peters *et al.*, 2006; Loparev *et al.*, 2007). However, no in-depth analysis of crossover history at the whole genome level has, to my knowledge, been reported previously.

For the work reported in this paper, it was of particular significance (and a powerfully simplifying circumstance for analysis of deep relationships) that examination of SNP patterns identified the same major groups as had earlier emerged from tree-building. This indicated that the incidence of recombination has been sufficiently low relative to that of nucleotide substitution to allow development of distinct lineages, at least for the U_L_ region, which represents close to 90 % of non-repeated genomic sequence and contributed 87 % of total SNPs in the dataset. Nonetheless, it is clear that genomes constituting recombinant forms between two distinct lineages have arisen. The analysis of possible crossover points described in this paper depended on optimizing continuity of SNP associations over groups, and then locating breakpoints in such associations. The crossovers that were clearly demonstrated in this way are supported by runs of SNPs which are sizeable with respect to overall diversity, and there are also other possible crossover events which are indicated by only one or two SNPs.

Sequence evolution in the S region, especially R_S_, appears to be more dynamic than that in U_L_. The incidence of SNPs in R_S_ is 1.5 times higher than in U_L_ (as seen in Fig. 1b[Fig f1]), and the occurrence of SNPs that define diversity within each multi-member group is more marked in both R_S_ and U_S_ than in U_L_ (Fig. 2a[Fig f2]). R_S_ is also distinctive in that its G+C content is much higher than that of the unique sequences (59 %, versus 44 and 43 % for U_L_ and U_S_, respectively). Such elevated G+C contents are a general feature of herpesviral genomic repeat regions, and are considered likely to result from mutations introduced or fixed during processes of recombination between repeat copies, perhaps involving biased gene conversion (McGeoch *et al.*, 1986, 2008). I interpret the S region's SNP pattern as probably indicative of higher levels, relative to U_L_, of both point mutation and recombination; since the SNP sets for R_S_ and U_S_ are small, no further analysis was attempted. It is worth noting that, in crossovers between distinct VZV strains, events involving R_S_ could well be a favoured class, given the recombinational activity between R_S_ copies in single-strain infection.

Studies of VZV geographical variation have been carried out with strategies that sample genome sequences (for instance Barrett Muir *et al.*, 2002; Quinlivan *et al.*, 2002), but available whole genome sequences apparently represent predominantly isolates from Caucasian populations (North American and European). It is striking that two deep lineages (J and M1) are presently represented by just one genome sequence each. The pOKA strain (parent of the OKA vaccine) which constitutes the J group is of Japanese origin, and may be representative of a still largely unexplored subset of VZV strains. It is also noteworthy that no complete VZV genome sequence, to the best of my knowledge, is considered to represent a sub-Saharan African isolate. Taking all into account, it is quite likely that further, uncharacterized deep lineages of VZV exist.

The diversity of VZV genomic sequences is recognized as being low compared with that seen in other human herpesviruses (Quinlivan & Breuer, 2006). For instance, diversity among genome sequences of herpes simplex virus type 1 is some sixfold higher than that in VZV (results not shown). The low diversity of VZV could result from one or both of two factors: lower rate of genomic change and lesser elapsed time since the most recent common ancestor of the characterized strains (discussed by  Barrett Muir *et al.*, 2002). Addressing such issues may become more feasible with the outline understanding we now have of lineages and recombination.

## Supplementary Material

[Supplementary Material]

## Figures and Tables

**Table 1. t1:** Complete genome sequences of VZV strains

**Strain**	**GenBank accession no.**	Group*
Dumas	X04370	E1
MSP	AY548170	E1
SD	DQ479953	E1
Sveta	EU154348	E1
KEL	DQ479954	E1
32p5	DQ479961	E1
NH293	DQ674250	E1
BC	AY548171	E1
36	DQ479958	E1
49	DQ479959	E1
11	DQ479955	E2
HJO	AJ871403	E2
22	DQ479956	E2
03500	DQ479957	E2
DR	DQ452050	M2
8	DQ479960	M2
pOKA	AB097933	J
CA123	DQ457052	M1

*After Loparev *et al.* (2007).
