# Supplementary material for: Lineages of varicella-zoster virus
Source: J Gen Virol. 2009 Apr;90(Pt 4):963–9. doi: 10.1099/vir.0.007658-0 (PMC2885040; doi:10.1099/vir.0.007658-0)
Supplement: [Supplementary Material] [file supp_90_4_963__index.html]

 Lineages of varicella-zoster virus -- McGeoch 90 (4): 963 Data Supplement - Supplementary Material -- Journal of General Virology

### Lineages of varicella-zoster virus, by Duncan J. McGeoch

*Journal of General Virology* vol. **90**, part 4, pp. 963–969

**Supplementary Material.** Supplementary methods, describing the production of the optimized colouring schemes for VZV UL SNPs, tables and figures are provided as a single PDF. [PDF] (391 KB)

  
  
