# Supplementary material for: Lineages of varicella-zoster virus
Source: J Gen Virol. 2009 Apr;90(Pt 4):963–9. doi: 10.1099/vir.0.007658-0 (PMC2885040; doi:10.1099/vir.0.007658-0)
Supplement: [Supplementary Material] [file supp_90_4_963__1.pdf]

Supplementary Methods

Optimized colouring schemes for VZV U_L SNPs

The aim of this exercise was to achieve a presentation of the VZV cross-group SNP set as a two-colour array that optimizes visible continuity of lineages, with the intention of using the resulting patterns of continuity to identify likely crossovers between lineages.

The five groups were taken as the objects of analysis rather than individual strains. The starting point was the 109 cross-group SNP set coloured with respect to the Dumas sequence (E1 group), and the analysis focused on U_L, excluding the 10 SNPs in the S region. Supplementary Fig. S1 shows the array of 99 U_L SNPs in binary coded form, with SNPs 17, 18 and 50 as asterisks in groups where they are variable. Each SNP locus possesses two of one character (0 or 1) and three of the other. There are ten possible arrangements of 2+3 characters (each of which could be written in two ways, e.g. 00111 or 11000). These patterns occur with the frequencies (excluding loci with asterisks) shown in Supplementary Table S1.

Supplementary Table S1.

Pattern no.	E1	E2	M2	J	M1	Number
1	0	0	1	1	1	28
2	0	1	0	1	1	12
3	0	1	1	0	1	0
4	0	1	1	1	0	0
5	0	0	0	1	1	1
6	0	0	1	0	1	11
7	0	0	1	1	0	24
8	0	1	0	0	1	17
9	0	1	0	1	0	3
10	0	1	1	0	0	0

The most frequent patterns by far are 1 and 7. These differ only in the state of group M1, and together account for 52 of the 96 loci. This suggested proceeding by putting M1 aside, to give four groups, for which there are only three distinct patterns possible for 2+2 characters (Supplementary Table S2).

Supplementary Table S2

Pattern	E1	E2	M2	J	Number
a	0	0	1	1	52
b	0	1	0	1	15
c	0	1	1	0	0

In addition, 29 of the SNP loci appear as singletons in the absence of group M1 (Supplementary Table S3).

Supplementary Table S3

Pattern	E1	E2	M2	J
w	0			
x		17		
y			11	
z				1

The bottom line of Supplementary Fig. S1 presents the abc and xyz codings as defined in Supplementary Tables S2 and S3. From inspection, reversing the coding (interchanging 1 and 0) at x loci improves coding continuity with adjoining loci (over the five groups) in all cases except SNPs 29 and 30, as shown in Supplementary Fig. S2. Next, reversing coding at b loci in the region of SNP 25 to SNP 33, with x loci at 29 and 30, plus b locus at SNP 79 also improves continuity with adjoining loci, as shown in Supplementary Fig. S3. Additionally reversing b loci at SNP 47 to SNP 54 is effectively neutral (Supplementary Fig. S4). Changing coding for y and z loci does not improve continuity.

This process yielded the codings of Supplementary Figs S3 and S4 as two versions of colouring scheme for U_L cross-group SNPs that optimize overall continuities of lineages. In the starting scheme (Supplementary Fig. S1) there were 95 changes of colour along the U_L SNPs (averaging for the two versions in E2 and M2 and then summing over the groups). In both optimized schemes the total was reduced to 65. It is to be emphasized that this was an empirical procedure, which may not be mathematically rigorous or find a unique set of solutions.

```
        1                                                 50
  E1    00000000000000000000000000000000000000000000000000
  E2    0000010000000000**0011011111111111111110110000111*
  M2    1111101111111111111100100000000000000001001111000*
   J    11111010011111111111001011110011100000010011111111
  M1    00111111111001111100110100011111111111101100101111
Code    aaaaaxayyaaaaaaa**aaxxaxbbbbxxbbbxxxxxxaxxaaaabbb*

        51                                              99
  E1    0000000000000000000000000000000000000000000000000
  E2    1111000000000000000010100001100000000000000000000
  M2    0000111110111111111101011110011111111111111111111
   J    1111110101101111100101010110111110011111011111111
  M1    1111101111111111111110111001111111110011000000000
Code    bbbbaayayzayaaaaayyaxaxayaaxbaaaayyaaaaayaaaaaaaa
```

Supplementary Fig. S1. Starting version: reference is E1.

```
        1                                                 50
  E1    00000100000000000000110100000000000111111011000000 00
  E2    0000000000000000**00000011111111100000000000000111*
  M2    1111111111111111111111110000000001111111111111000*
   J    11111110011111111111111111110011111111111111111111
  M1    00111011111001111100000000011111100000000000101111
Code    aaaaaxayyaaaaaaa**aaxxaxbbbbxxbbbxxxxxxaxxaaaabbb*

        51                                              99
  E1    0000000000000000000010100001000000000000000000000
  E2    1111000000000000000000000000100000000000000000000
  M2    0000111110111111111111111111011111111111111111111
   J    1111110101101111100111110111111110011111011111111
  M1    1111101111111111111100011000111111111100110000000
Code    bbbbaayayzayaaaaayyaxaxayaaxbaaaayyaaaaayaaaaaaaa
```

Supplementary Fig. S2. Reverse coding in x columns except at columns 29 and 30.

```
        1                                                50
  E1    00000100000000000000110111111111111111101100000000
  E2    0000000000000000**0000000000000000000000000000111*
  M2    1111111111111111111111111111111111111111111111000*
   J    11111110011111111111111100001100011111111111111111
  M1    00111011111001111100000011100000000000000000101111
Code    aaaaaxayyaaaaaaa**aaxxaxbbbbxxbbbxxxxxxaxxaaaabbb*

        51                                              99
  E1    0000000000000000000010100001100000000000000000000
  E2    1111000000000000000000000000000000000000000000000
  M2    0000111110111111111111111111111111111111111111111
   J    1111110101101111100111110111011110011111011111111
  M1    1111101111111111111100011000011111111100110000000
Code    bbbbaayayzayaaaaayyaxaxayaaxbaaaayyaaaaayaaaaaaaa
```

Supplementary Fig. S3. Reverse coding in some b and remaining x columns.

```
        1                                                50
  E1    00000100000000000000110111111111111111101100001111
  E2    0000000000000000**0000000000000000000000000000000*
  M2    1111111111111111111111111111111111111111111111111*
   J    11111110011111111111111100001100011111111111110000
  M1    00111011111001111100000011100000000000000000100000
Code    aaaaaxayyaaaaaaa**aaxxaxbbbbxxbbbxxxxxxaxxaaaabbb*

        51                                              99
  E1    1111000000000000000010100001100000000000000000000
  E2    0000000000000000000000000000000000000000000000000
  M2    1111111110111111111111111111111111111111111111111
   J    0000110101101111100111110111011110011111011111111
  M1    0000101111111111111100011000011111111100110000000
Code    bbbbaayayzayaaaaayyaxaxayaaxbaaaayyaaaaayaaaaaaaa
```

Supplementary Fig. S4. Alternative to Supplementary Figure S3: reverse coding in all b and remaining x columns.
